# Supplementary material for: Identification of photoperiod-induced specific miRNAs in the adrenal glands of Sunite sheep (Ovis aries)
Source: Front Vet Sci. 2022 Jul 22;9:888207. doi: 10.3389/fvets.2022.888207 (PMC9354845; doi:10.3389/fvets.2022.888207)
Supplement: Supplementary file 1 [file Data_Sheet_1.ZIP › Supplementary file/miRDeep2 file.zip!/miRDeep2 file/Know_Structure/SPLP42A3/oar-mir-411b.pdf]

miRBase precursor : oar-mir-411b
Total read count : 1747
oar-miR-411b-5p read count : 1714
oar-miR-411b-3p read count : 32
remaining reads : 1

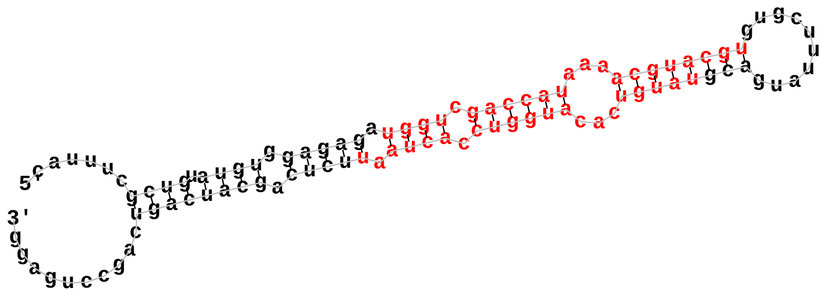

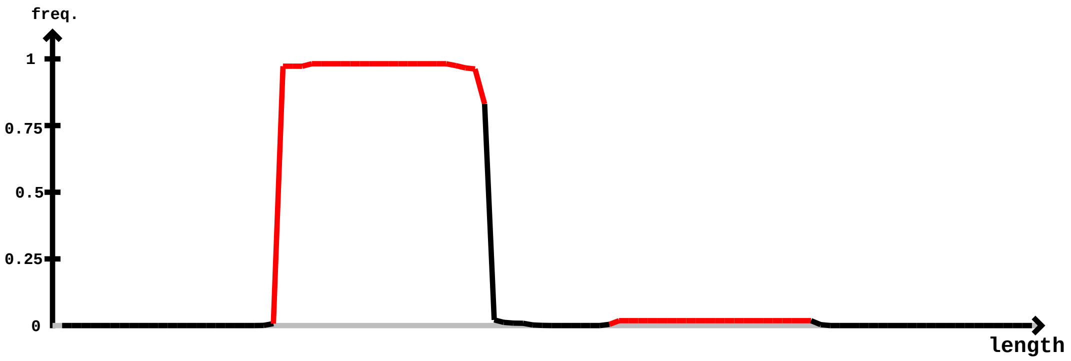


```
                                                           oar-miR-411b-3p
                      oar-miR-411b-5p
5'- cauuucgcuguauguggagagauggucgaccauaaaacguacgugugcuuuaugacguaugucacaugguccacuaauucucagcaucagucagccugagg -3'   exp
    ......((((.((((.(((((.((((.((((((...((((((((..........))))))))...)))))).)))).))))).))))))))..........   reads   mm   sample
    ....................gauggucgaccauaaaacguacg..........................................................   1       0    seq
    .....................auggucgaccauaaaacguacg..........................................................   10      0    seq
    .....................auggucgaccauaaaacguacgu.........................................................   2       0    seq
    ......................uggucgaccauaaaacgu.............................................................   12      0    seq
    ......................uggucgaccauaaaacgua............................................................   15      0    seq
    ......................uggucgaccauaaaacguac...........................................................   7       0    seq
    ......................uggucgaccauaaaacguacg..........................................................   219     0    seq
    ......................uggucgaccauaaaacguacgu.........................................................   1412    0    seq
    ......................uggucgaccauaaaacguacgug........................................................   16      0    seq
    ......................uggucgaccauaaaacguacgugu.......................................................   5       0    seq
    .........................ucgaccauaaaacguacgugug......................................................   1       0    seq
    .........................ucgaccauaaaacguacgugugc.....................................................   11      0    seq
    .........................ucgaccauaaaacguacgugugcu....................................................   3       0    seq
    .........................ucgaccauaaaacguacgugugcuu...................................................   1       0    seq
    ........................................................guaugucacaugguccacuaau.......................   7       0    seq
    ........................................................guaugucacaugguccacuaauu......................   1       0    seq
    .........................................................uaugucacaugguccacuaau.......................   19      0    seq
    .........................................................uaugucacaugguccacuaauu......................   5       0    seq
```
